# Isolated Pancreatic Agenesis Secondary to PTF1A Gene Mutation: A Case Series and Literature Review

**DOI:** 10.7759/cureus.47202

**Published:** 2023-10-17

**Authors:** Afaf I Alsagheir, Angham AlMutair, Sarah Bakhamis, Lujain Aletani, Shahad Alhumaidi, Bassam Bin Abbas

**Affiliations:** 1 Department of Pediatrics, Division of Endocrinology, King Faisal Specialist Hospital and Research Centre, Riyadh, SAU; 2 Department of Pediatrics, Division of Endocrinology, King Abdulaziz Medical City, King Abdullah Specialist Children’s Hospital, Ministry of National Guard-Health Affairs, Riyadh, SAU; 3 Department of Pediatrics, Section of Pediatric Endocrinology, King Khalid University Medical City, Abha, SAU

**Keywords:** saudi arabia, monogenic diabetes, ptf1a mutation, pancreatic agenesis, neonatal diabetes

## Abstract

Background

Neonatal diabetes mellitus is a rare form of monogenic diabetes which is diagnosed in the first six months of life. It is often related to genetic mutations; hence, genetic testing is warranted. Here, we present six cases of pancreatic agenesis resulting in neonatal diabetes with *PTF1A *gene mutation.

Methodology

This retrospective case series study included six pediatric cases of neonatal diabetes mellitus who are currently following at pediatric endocrinology clinics at King Faisal Specialist Hospital and Research Center, Riyadh, Saudi Arabia.

Results

The study reported six patients with a mean age of eight years who presented with pancreatic agenesis resulting in neonatal diabetes with *PTF1A *gene mutation. In four patients, there was no evidence of cerebellar agenesis.

Conclusions

Neonatal diabetes is a challenging disease that must be diagnosed early to prevent subsequent metabolic complications. Genetic testing is recommended in neonates who present with prolonged duration of hyperglycemia. Insulin replacement is the treatment of choice.

## Introduction

Neonatal diabetes is a type of diabetes that occurs due to an underlying monogenic effect [1]. It affects 1 in every 90,000-160,000 live births [2]. Neonatal diabetes can be categorized into a transient or a permanent form with more than 20 reported gene mutations [1]. One of the known causative gene mutations is the *pancreas transcription factor 1α* (*PTF1A*) gene defect [3]. It is known to play a crucial role in pancreatic and cerebellar development [4]. All reported cases with this mutation did not survive more than four months [4-7]. A rare coding hypomorphic mutation in the *PTF1A* gene was reported in four individuals from two different families who had the same novel mutation, p.P191T, and presented with permanent neonatal diabetes (PNDM) without cerebellar manifestations [8]. Isolated pancreatic agenesis was related to the biallelic mutations in an enhancer located near the *PTF1A* gene; this phenotype suggests that the enhancer is pancreas-specific [8]. Patients with *PTF1A *enhancer mutation presented with severe PNDM and pancreatic exocrine insufficiency without neurological involvement or other extrapancreatic features [7-10]. Recently, Demirbilek et al. reported the largest series of 30 patients with five different homozygous mutations in *PTF1A *enhancer. They expanded the phenotypic presentation to include anemia, cholestasis, and impaired growth, along with PNDM and exocrine pancreatic insufficiency [11]. *PDX1 *is another transcription factor gene in which biallelic mutation resulted in isolated pancreatic agenesis presented with permanent neonatal diabetes and variable degrees of exocrine pancreatic [5,12,13]. Isolated PNDM without exocrine insufficiency may result from a hypomorphic mutation in *PDX1 *[14,15].

In this study, we report six patients from four families, five of whom presented with PNDM and exocrine pancreatic insufficiency without cerebellar pathology and one with cerebral agenesis. They were found to have the same rare *PTF1A *coding hypomorphic mutations. This is the third time that this mutation has been reported worldwide.

## Materials and methods

Human subjects

This retrospective case series study involved six pediatric cases of neonatal diabetes mellitus who are currently following at pediatric endocrinology clinics at King Faisal Specialist Hospital and Research Center (KFSH&RC), Riyadh, Saudi Arabia. Data were collected from our database in July 2023. The study included patients aged less than 14 years with neonatal diabetes mellitus. Patients with no available genetic testing were excluded from the study. Patients’ demographics, history, presentations, and investigations were recorded.

This study was approved by the Office of Research Affairs at King Faisal Specialist Hospital and Research Centre (reference number: 2231153).

Genetic testing

Genetic testing was done during routine clinical practice. After obtaining patient consent, DNA was extracted from the peripheral blood samples, and whole exome sequencing was done at the Molecular Diagnostic Laboratory of the Clinical Genomic Department Center for Genomic Medicine at KFSH&RC.

## Results

Case one

A 10-year-old male proband on family 1(1-1) was diagnosed on day six with diabetes and congenital hypothyroidism. He was born full-term by cesarean section due to fetal distress. His birth weight was 2.6 kg. Clinically, he had no dysmorphic features. He was the second-born child of first-cousin Saudi parents. There was no family history of neonatal deaths, abortion, or any other morbidities. He was in the hospital for six months after delivery due to brittle diabetes with no ketosis. He was also started on pancreatic enzyme at three months of life as he was found to have malabsorption with poor weight gain and elevated fecal fat, leading to a suspicion of exocrine pancreatic insufficiency. He initially had anemia, which required multiple transfusions but improved after the first year of life.

Laboratory evaluation at diagnosis showed a random insulin level of 7 pmol/L (12-150 pmol/L), C-peptide level of 0.1 nmol/L (0.26-0.62 nmol/L), amylase level of 31 U/L (23-85 U/L), lipase level of 4 U/L (0-16 U/L), and elevated liver enzymes (alanine transaminase (ALT) 149 U/L (7-55 U/L), aspartate transaminase (AST) 133 U/L (8-48 U/L), and gamma-glutamyltransferase (GGT) 375 U/L (0-30 U/L)). Free T4 level was 7 pmol/L (12-22 pmol/L), and thyroid-stimulating hormone (TSH) level was 65 mU/L (0.4-4 mUL).

Genetic testing showed *PTF1A* homozygous mutation (c.5171C>A p.(Pro191Thr). MRI of the abdomen did not identify any pancreatic tissue. The liver, gallbladder, spleen, and the rest of the gastrointestinal imaging were unremarkable. MRI of the brain was also unremarkable. The echocardiogram was normal.

He was managed with subcutaneous insulin (Aspart and Degludec), pancreatic enzymes, and L-thyroxine. He continued to have hyperglycemia, requiring multiple daily injections of insulin. His hemoglobin A1c is currently ranging from 7.95 to 10.3% with normal liver enzymes and hemoglobin. He was growing optimally after the first year of life with no other complications and no neurological symptoms.

Case two

The patient was a three-month-old younger brother of a previous patient who was also identified as having neonatal diabetes. The baby was delivered at full term by normal vaginal delivery and was diagnosed with intrauterine growth restriction with a birth weight of 1.6 kg. He was admitted to the intensive care unit for 15 days due to persistent hyperglycemia of more than 250 mg/dL and hemoglobin A1c of 11.2%. His laboratory investigations showed normal thyroid screening, a low random insulin level of 7 pmol/L (12-150 pmol/L), and a low C-peptide level of 0.5 nmol/L (0.26-0.62 nmol/L). Furthermore, his abdominal ultrasound showed a small pancreas with the head measuring 0.7 cm, the body measuring 0.3 cm, and no visualization of the tail. His head ultrasound showed grade 2 intraventricular hemorrhage with no signs of hydrocephalus. His genetic testing showed a *PTF1A* homozygous mutation (c.5171C>A p.(Pro191Thr) as his brother, following which he was started on subcutaneous insulin accordingly. Soon after discharge, he presented to the emergency department complaining of cough, fever, and dehydration. He was admitted with a case of diabetic ketoacidosis with a glucose level of 350 mg/dL and a bicarbonate level of 13. He had a chest infection associated with COVID-19. His chest infection was followed by acute respiratory distress syndrome and multiorgan failure at the age of three months which led to his death.

Case three

A 17-year-old boy was born at term to consanguineous parents with a birth weight of 2 kg. He presented on the first day of life with hyperglycemia not related to stress or medications. There were no other neurological or dysmorphological manifestations. At the age of four days, a diagnosis of neonatal diabetes was made and subcutaneous insulin was adjusted based on his needs. His initial investigation showed a glucose level of 210 mg/dL and hemoglobin A1c of 12%. Throughout his clinical course, he was evaluated for short stature and failure to thrive. Celiac screening was positive and was started on a gluten-free diet. Other laboratory investigations were normal, including renal, electrolyte profile, thyroid function test, and growth hormone on stimulation. Random insulin levels and C-peptide levels were repeatedly low. Amylase level was 18 U/L (23-85 U/L), and lipase level was 2 U/L (0-16 U/L). He continued to exhibit poor weight gain despite appropriate insulin dosing and a gluten-free diet, associated with recurrent complaints of foul-smelling diarrhea, and required ADEK vitamin replacement. Exocrine pancreatic insufficiency was suspected and confirmed by elevated fecal fat. Exocrine pancreatic enzyme replacement therapy was started at the age of 11 years. He underwent a detailed neurodevelopmental assessment by a neurodevelopmental pediatrician, and the evaluation revealed normal neurological examination apart from searching eye movements with horizontal nystagmus. Abdominal ultrasonography failed to visualize the pancreas. Further evaluation by abdominal MRI showed no visualization of the pancreas within its expected location which is compatible with pancreatic agenesis. Brain MRI showed normal brain structure with no acute intracranial insult. Whole-exome sequencing showed a *PTF1A *homozygous mutation (c.5171C>A p.(Pro191Thr). The echocardiogram was normal.

Case four

A 14-year-old girl, the younger sister of case three, was born at term with a birth weight of 2.3 kg. She was diagnosed with neonatal diabetes on the second day of life and started on insulin therapy. Insulin doses were adjusted based on her requirements. Clinically, she had no dysmorphism or neurological abnormalities. Initial investigation showed a glucose level of 220mg/dL and hemoglobin A1c of 8.8%. Her insulin and C-peptide levels were 6 pmol/L and 0.3 nmol/L, respectively. She also complained of poor weight gain and steatorrhea, with an elevated fecal stool, which led to a diagnosis of exocrine pancreatic insufficiency. She was started on replacement therapy at the age of eight years. The patient underwent a detailed neurodevelopmental assessment by a neurodevelopmental pediatrician, and the evaluation revealed a normal neurological examination. Abdominal MRI showed no visualization of the pancreas within its expected location which is compatible with pancreatic agenesis. Brain MRI showed normal brain structure with no acute intracranial insult. Whole-exome sequencing showed a *PTF1A* homozygous mutation (c.5171C>A p. (Pro191Thr). The echocardiogram was reported normal.

Case five

A two-year-old boy was born at full term via vaginal delivery. Hyperglycemia was noticed on the third day of life and persisted above 300 mg/dL. Insulin level was <2.7 pmol/L (12-150 pmol/L) and C-peptide level was 0.07 nmol/L (0.26-0.62 nmol/L). Subcutaneous short-acting insulin was started according to a sliding scale, in addition to long-acting basal insulin (the patient had brittle diabetes with fluctuations in his blood glucose readings, which was difficult to control. Multiple insulin regimens were tried. Currently, the patient is on insulin Digludec 0.5 U/kg once daily only, and rapid-acting insulin 0.5-1 U is required occasionally if blood glucose is above 250 mg/dL before meals).

**Figure 1:**
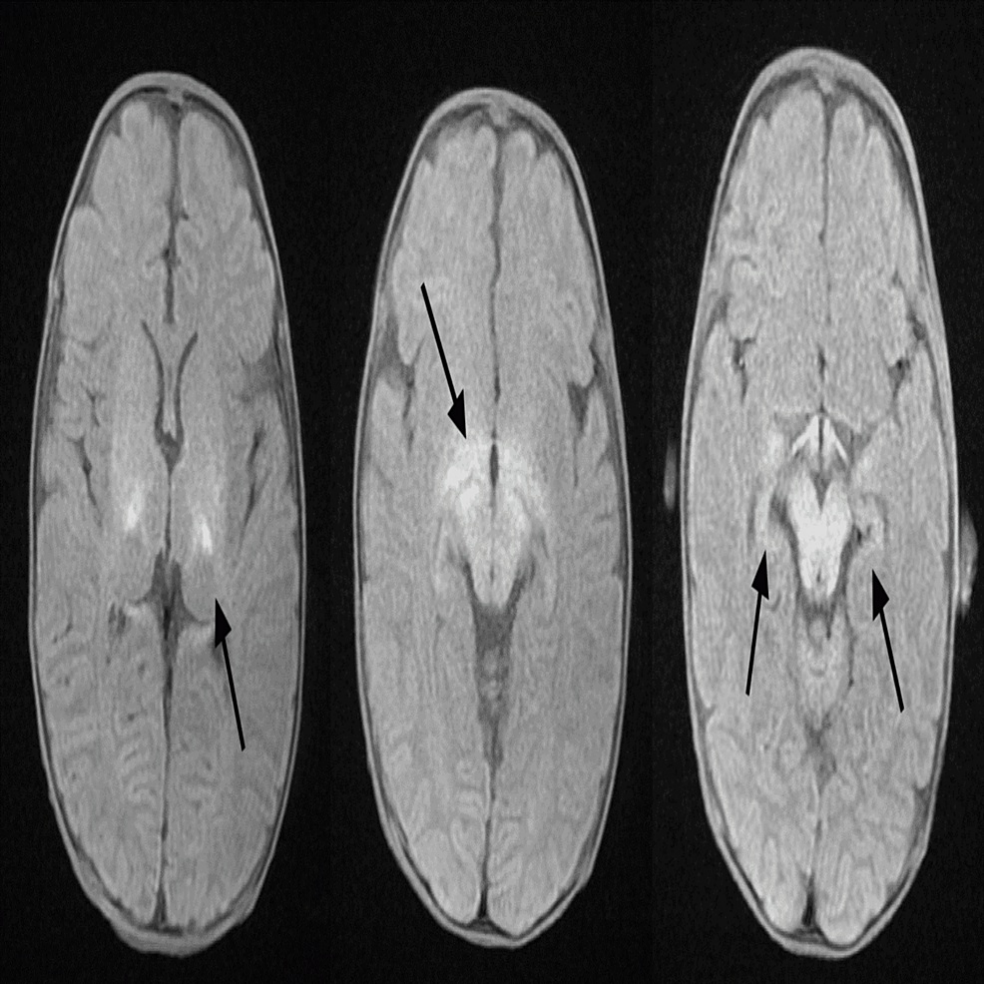
MRI of the brain showing abnormal signal intensity involving the ventral lateral thalami and subthalamic nuclei which was compatible with hyperglycemic encephalopathy (see black arrows).

The child had severe failure to thrive which was managed by a high caloric intake formula through a gastrostomy tube with no improvement. Pancreatic insufficiency was suspected due to the development of malabsorption and elevated fecal fat. Pancreatic enzyme replacement was initiated at three months of age. The patient had a profound global developmental delay with seizure, irritability, and hypotonia. Phenobarbital was started on day three of life. MRI of the brain showed abnormal signal intensity involving the ventral lateral thalami and subthalamic nuclei, which was compatible with hyperglycemic encephalopathy (Figure 1). An echocardiogram showed tiny aorta pulmonary collateral with an ejection fraction of 69%. On CT, there were no intrathoracic collaterals, and no intervention was needed.

Laboratory evaluation showed persistent anemia, high transaminase, hypoalbuminemia, and metabolic acidosis (evaluated but all investigations were normal, resolved at the age of nine months). Renal ultrasound showed bilateral medullary nephrocalcinosis.

Whole-exome sequencing showed a *PTF1A *homozygous mutation c.5171C>A p.(Pro191Thr) which confirmed the diagnosis of pancreatic agenesis

Case six

A five-year-old girl born full-term via vaginal delivery was diagnosed with intrauterine growth restriction with a birth weight of 1.9 kg. Clinically, she had no dysmorphism and no neurological abnormalities, and the rest of the physical examination was unremarkable.

Hyperglycemia was noticed on day four of life and persisted above 300 mg/dl. Insulin level was <2.7 pmol/L (12-150 pmol/L) and C-peptide level was 0.2 nmol/L (0.26-0.62 nmol/L). The pancreas was not visualized on an MRI of the abdomen. Subcutaneous short-acting insulin was started according to a sliding scale in addition a long-acting basal insulin. Pancreatic insufficiency was suspected due to the development of steatorrhea, and pancreatic enzyme replacement was initiated. Laboratory evaluation showed anemia (hemoglobin of 6-7 g/L in the first six months but improved later), normal thyroid function, and normal liver enzymes. Whole-exome sequencing showed a *PTF1A* homozygous mutation c.5171C>A p.(Pro191Thr).

Currently, the patient is growing optimally on Insulin Aspart and Insulin Degludec at 0.9 U/kg/day. The latest hemoglobin A1C is 10%, and there are no other concerns.

Table 1 presents the baseline data of all included patients, and Table 2 presents the laboratory investigation findings of all included patients.

**Table 1:** Baseline data of included cases with PTF1A (n = 6). FAM1-1: family 1 proband 1; FAM1-2: family 1 proband 2; FAM3-1: family 3 proband 1; FAM4-1: family 4 proband 1; DM: diabetes mellitus; PNDM: permanent neonatal diabetes; BMI: body mass index

Family	Age	Gender	Birth weight	Age at diagnosis with DM in (days)	Presence of PNDM/DM	Pancreas agenesis/hypoplasia (MRI pancreas)	Exocrine pancreas insufficiency	Cholestasis elevated transaminases	Anaemia	Growth retardation at follow-up	Neurological symptoms	Hypothyroidism	Latest height/length (SDS)	Latest BMI (kg/m^2^)	Current insulin dose (U/kg/day)
FAM1-1	10 years	Male	2.6 kg	Day 6	Yes	Pancreas not visualized on MRI	Yes	Yes	Yes	No	No	Yes	-0.9	16	0.8
FAM1-2	3 months	Male	1.5 kg	Day 1	Yes	Small Pancreas , no visualization of the tail	Yes	No	Yes	Yes	Grade 2 intraventricular haemorrhage on US head		-3.5	Weight/Length -1.5 SD	0.3
FAM2-1	17 years	Male	2 kg	Day 4	Yes	Pancreas not visualized on MRI	Yes	No	Yes	No	No	No	-0.85	26.6	1.5
FAM2-2	14 years	Female	2.6 kg	Day 2	Yes	Pancreas not visualized on MRI	Yes	No	Yes	No	No	NO	-1.12	19	1.1
FAM3-1	2 years	Male	1.3 kg	Day 2	Yes	Pancreas not visualized on MRI	Yes	No	Yes	Yes	Microcephaly, global developmental delay, and epilepsy	No	-8.85	Weight/Length -2.5 SD	0.5
FAM4-1	5 years	Female	2.2 kg	Day 12	yes	Pancreas not visualized on MRI	Yes	No	Yes	No	No	No	-1.5	13	0.9

**Table 2:** Laboratory investigations (n = 6). FAM1-1: family 1 proband 1; FAM1-2: family 1 proband 2; FAM3-1: family 3 proband 1; FAM4-1: family 4 proband 1; ALT: alanine transaminase; AST: aspartate transaminase; GGT: gamma-glutamyltransferase; HbA1C: hemoglobin A1C

Family	Hemoglobin (125–155 g/L	Serum insulin (12–150 pmol/L)	C-peptide (0.26–0.62 nmol/L)	HbA1c	ALT (5–55 U/L)	AST (5–34 U/L)	GGT (12–64 U/L)	Albumin (38–54 g/L)	Amylase (0–60 IU/L )	Lipase (30–110 IU/L)	Bilirubin (3.4–20.5 µmol/L)	Fecal fat screen
FAM1-1	86	7 pmol/L	0.1	9%	149	133	375	16	31	4	145.9	>60 fat globules seen
FAM1-2	74	7 pmol/L	0.5	11.20%	34	28	21	38	Not done	Not done	6	>60 fat globules seen
FAM2-1	73	Not done	Not done	12%	23	28	45	34	18	2	10	>60 fat globules seen
FAM2-2	81	23	0.3	10.50%	54	49	39	39	16	5	13	>60 fat globules seen
FAM3-1	72	<2.78	0.07	9.50%	47	37	23	35	Not done	Not done	7	>60 fat globules seen
FAM4-1	83	12	0.2	10%	67	54	47	19	10	6	17	>60 fat globules seen

## Discussion

Diabetes mellitus is a metabolic disease that is caused by a group of environmental, biological, and genetic factors [1]. Neonatal diabetes is considered to be of genetic cause and may occur solely or concurrent with other syndromes [16,17]. The difference between transient and PNDM is that the transient picture of the disease remits and relapses several years after while the latter stays and does not remit [1]. PNDM has been reported to be associated with several gene mutations including *PTF1A*, *GLIS3*, *PDX1*, *NEUROD1*, and *HNF1B *[1].

Pancreatic agenesis has been linked before to neonatal diabetes. In 1993, Wright et al. made the first connection between neonatal diabetes and pancreatic agenesis [5]. However, it was not until 2009 that a link between various genetic markers, such as *PTF1A*, and neonatal diabetes was made in a Turkish newborn infant [9]. Later, in 2010, Balasubramanian et al. reported three cases of PNDM that had normal phenotypes with no signs of mutation in several genes including the *PTF1A *[10]. However, the three patients had pancreatic hypoplasia rather than agenesis which differed from the *PTF1A* gene mutation cases. In the same year, D'Amato et al. reported PNDM with pancreatic agenesis associated with congenital heart anomalies [11]. However, the genetic screening panel found the mutation to be in *GATA4 *rather than *PTF1A *or *PDX-1*, which are linked to the development of the pancreas [11]. Catli et al. reported a case of a 40-day-old male infant who was diagnosed with PNDM. The patient had congenital heart defects along with pancreatic agenesis. His phenotype showed mutations in the *GATA6* gene with no evidence of exocrine pancreas insufficiency [12]. In 2014, Shalev et al. described the triad of microcephaly, epilepsy, and neonatal diabetes due to compound heterozygous mutations in the *IER3IP1* gene [18]. Similar to our siblings, Gonc et al. reported two siblings with neonatal diabetes who had a homozygous *PTF1A* enhancer mutation, with one diagnosed early during infancy, while the older sister had a milder form with onset starting at the age of nine [19]. Gabbay et al. in 2017 further analyzed the *PTF1A *mutation which was found in their reported neonatal diabetes case. They found a frameshift mutation in exon 1 (c.437_462 del, p.Ala146Glyfs*116) and a mutation affecting a highly conserved nucleotide within the distal pancreatic enhancer (g.23508442A>G), highlighting more possible mutations with further genetic studies needed [20]. Kurnaz et al. also found a homozygous *PTF1A *enhancer mutation in two infants who were treated with a conventional insulin pump. Both neonates had hyperglycemia in the first month of life of more than 250 mg/dL making subcutaneous insulin an ideal treatment for such cases of pancreatic agenesis causing PNDM [13]. Lastly, in 2018, Evliyaoğlu et al. reported three cases of neonatal diabetes, with two having isolated pancreatic agenesis due to *PTF1A *mutation, while the third neonate developing epilepsy and developmental delay alongside pancreatic agenesis with *KCNJ11 *mutation [21].

De Franco et al. studied neonatal diabetes in a cohort study and reported that neonatal diabetes is linked to 16 syndromes with 17 different gene mutations [22]. Compared to our two cases, the first case reported a gene mutation of *PTF1A *without any mutation in *GLIS3*. Furthermore, pancreatic agenesis was only present without cerebellar agenesis as well as congenital hypothyroidism. Similarly, the second case only reported pancreatic agenesis without cerebellar with a reported gene mutation of *PTF1A*.

Barbetti et al. reported that the prevalence of neonatal diabetes is high in countries where parental consanguinity is high [23]. Other risk factors include Wolfram syndrome and a family history of hyperinsulinemia. Our reported cases reported consanguinity with no family history of diabetes or genetic syndromes. The diagnosis of neonatal diabetes should be suspected in low birth weight infants with persistent high blood glucose levels of more than 250 mg/dL [22,23]. Early treatment of neonatal diabetes is crucial to prevent and achieve metabolic control as it is more challenging to control it than diabetes in the adult group. Brain development and cognitive development can be affected greatly by prolonged hyperglycemia as well as recurrent attacks of hyperglycemia [22,23]. At present, insulin therapy by subcutaneous injections is the treatment of choice in newborns with neonatal diabetes to treat and prevent ketoacidosis and dehydration [23]. In cases of persistent hyperglycemia despite insulin administration, prolonged hospitalization with insulin infusion might be required [23]. In all our cases, blood sugar levels were difficult to control during the first few months of life, patients were very sensitive to insulin, and hypoglycemia developed frequently. Diabetic ketoacidosis occurred only in the second patient who presented with a severe chest infection, and a history of prolonged hospitalization of two to six months was noted in all patients. Meanwhile, the death of the second patient was most probably due to a severe chest infection caused by COVID-19 which led to multiorgan failure. The rest of our patients are alive and doing well with no neurological abnormalities, except for the fifth patient who had severe global development delay. We are not sure if this is part of his condition or related to severe hyperglycemic episodes he was encountering during the neonatal period (Tables 1, 2).

We reported the same homozygous *PTF1A *mutation in six affected individuals from four Saudi families who likely inherited the mutation from a common distant ancestor, as this variant has previously been described by Houghton et al. [7] in one Saudi family and one Kuwaiti family.

This study has a few limitations. It is a retrospective study with a small number of reported cases. This is expected given the rarity of the disease, but because there is little information about it in the literature, it is crucial that we share our experiences with this unusual kind of diabetes.

## Conclusions

Neonatal diabetes is a challenging disease that must be diagnosed early to prevent subsequent metabolic complications. Genetic testing is recommended in neonates who present with prolonged duration of hyperglycemia. Insulin replacement is the treatment of choice. Our study has expanded the number of cases associated with hypomorphic *PTF1A *missense mutations, as well as improved the understanding of their phenotypes. With the previously reported cases and our cases, we anticipate a higher prevalence in the Arab region. This mutation should be considered in a patient presented with neonatal diabetes and no neurological symptoms.
